# MRI and CT imaging characteristics in parotid tumors with false-negative fine-needle aspirations

**DOI:** 10.1186/s13005-024-00467-5

**Published:** 2024-10-26

**Authors:** Hyun Jee Lee, Hee Jin Kang, Jong Hwan Lee, Min Kyeong Lee, Su Il Kim, Young Chan Lee, Young-Gyu Eun

**Affiliations:** 1 Department of Otolaryngology Head & Neck Surgery, Kyung Hee University School of Medicine, Kyung Hee University Medical Center, Seoul, Korea; 2 Department of Biomedical Science and Technology, Graduate School, Kyung Hee University, Seoul, Republic of Korea

**Keywords:** Parotid tumors, Fine-needle aspiration(FNA), Radiological imaging, Benign and malignant tumors, False-negative results

## Abstract

**Backgrounds:**

Preoperative imaging, particularly with magnetic resonance imaging (MRI) and computed tomography (CT) scans, plays a crucial role in distinguishing between benign and malignant parotid gland tumors, while the reliability of Ultrasound-Guided Fine Needle Aspiration (FNA) in diagnosing these masses remains a topic of debate.

**Methods:**

This two-center retrospective analysis was conducted on 347 patients with parotid gland tumors who had FNA and preoperative imaging (CT or MRI). All patients underwent surgery and final histopathological examination was available, along with complete medical records between January 2008 and May 2023.

**Results:**

Among the 347 patients, 318 (92%) had benign and 10 (3%) had malignant tumors based on FNA, with 19 (5%) unsatisfactory specimens. Final histological diagnosis revealed 303 (87%) benign and 44 (13%) malignant lesions, with a false-negative rate of 10.6% for FNA. Multivariate analysis identified irregular shape and invasion as independent predictors of malignancy in patient with benign or unsatisfactory FNA results. The odds ratio for irregular shape was 3.06 and for invasion was 12.73.

**Conclusion:**

Imaging characteristics, such as irregular shape and invasion may indicate towards malignant parotid tumors, even in patients with false-negative benign findings in FNA.

## Background and purpose

Preoperative characterization of parotid gland tumors using dedicated imaging is crucial for appropriate treatment planning because the surgical approach differs for benign and malignant lesions [1]. Several studies have explored the diagnostic accuracy of different modalities for parotid gland tumors. The role of imaging modalities such as magnetic resonance imaging (MRI) and computed tomography (CT) has been widely studied. Common indicators of benign parotid tumors include well-defined margins, round shape, homogeneous density on CT scans, and high signal intensity on T2-weighted MRI images [2, 3]. Instead, hypointensity on T2-weighted MRI images, irregular or poorly defined margins, and infiltration into surrounding tissue (parapharyngeal space, muscles, and bone) can be considered suspicious for malignancy [4, 5]. 

Fine-needle aspiration (FNA), a commonly used preoperative diagnostic tool, has shown variable accuracy rates. According to a study by Schmidt et al., FNA has a sensitivity of 96% and a specificity of 98% for diagnosing parotid gland tumors [6], while other studies report sensitivity as low as 66% and specificity 81% [7]. These discrepancies reveal the limitations of FNA in reliably differentiating between benign and malignant tumors. And the role of FNA in the preoperative investigation of parotid lesions remains controversial [6, 8]. 

The aim of this study was to evaluate specific MRI and CT imaging characteristics to improve the ability to distinguish between benign and malignant parotid tumors, particularly in cases where FNA results are false negative.

## Materials and methods

### Patient selection

This two-center retrospective study was approved by the ethics committee of our hospital. In this study, patients after a parotidectomy for a parotid gland tumor with a preoperative CT or MRI and FNA between January 2008 and May 2023 were eligible. All tumors were surgically resected and histologically classified. Exclusion criteria included patients with history of previous head and neck surgery or radiation therapy, a cancer diagnosis those who did not undergo fine-needle aspiration cytology before surgery, those who did not have imaging studies performed, or patients with incomplete medical records.

### Study design

Preoperative FNA results were compared with the final histological diagnoses of the surgical specimens. We divided the patients into two groups based on the results of preoperative FNA and the final histological examination after surgery. The true-negative group who had benign or unsatisfactory results on preoperative FNA and were confirmed to be benign on the final histological examination. The false-negative group who had benign or unsatisfactory results on FNA but were diagnosed with malignancy on the final histological examination.

We aimed to determine the preoperative imaging factors that predict malignancy on biopsy in patients with benign or unsatisfactory preoperative FNA results.

### Ultrasound-guided fine-needle aspiration cytology and final histologic diagnosis

All ultrasound-guided FNA procedures were performed by experienced otolaryngologists. Details of cytologic interpretation, including specimen quality, cell types identified, cytologic impression, specific cytologic diagnosis, and specific histologic diagnosis, were recorded by cytopathologists. According to the report by cytopathologists, we categorized into three types: (1) benign cytology, (2) malignant cytology, and (3) unsatisfactory cytology (inconclusive).

Surgical excision was performed by two head and neck staff members.

Based on the final histologic diagnosis of the surgical specimen, FNA specimens were categorized as follows (Appendix 1):

- True positive : cytologic diagnosis of malignancy consistent with histological findings;
- False positive : malignant cytology and benign histology;
- True negative : benign or unsatisfactory cytology, benign histology;
- False negative : benign or unsatisfactory cytology, malignant histology;

### Data collection

Demographic, clinical, cytopathology, and histopathological data were entered into a computer database. Details of the cytological interpretation, including specimen quality, identified cell types, cytological impression, specific cytological diagnosis, and specific histological diagnosis, were recorded.

### Image analysis

All imaging analyses were performed by two independent radiologists (subspecialized in head and neck) who were blinded to clinical information and histopathologic results. The location (superficial lobe, deep lobe, or whole lobe), multiplicity (single, two, more than two), laterality (unilateral or bilateral) and maximum diameter of tumors were measured on CT and MRI. We analyzed the following imaging parameters: shape (regular or irregular), margin (well defined, ill defined, infiltrative, or none), contrast enhancement (homogenous, heterogenous, subtle, or none), cystic contents, lobulated contour, invasion, and calcification.

### Statistical analysis

Statistical evaluations were performed using R version 4.3.1. Fisher’s exact test was performed to evaluate the radiologic relationship between benign and malignant tumors. Univariate and multivariate logistic regression analyses were performed. The regression model included the image characteristics of the covariates (enhancement, shape, margin, cystic, lobulated, invasion, and calcification). Odds ratios (ORs) were analyzed to determine the degree of influence of different factors such as age, sex, medical history (Diabetes, Hypertension), mass diameter, location, laterality, and multiplicity. Statistical significance was set at *p* < 0.05.

## Results

### Demographic & clinical characteristics

During the study period, 412 patients with parotid gland tumors underwent surgery. Patients with incomplete medical records were excluded. Among them, 347 patients (197 males, 150 females; median age, 55.7 years; range, 14–85 years) with parotid gland tumors who underwent FNA and imaging (MRI or CT) before surgery were included and retrospectively analyzed. Sixty-five patients had only CT, 261 patients had only MRI, and 21 patients had both tests.

Tumors were mainly located in the superficial parotid lobe (208 cases, 60%), whereas 27 (8%) and 112 (32%) tumors were located in the deep and whole parotid lobes, respectively. The laterality was unilateral in 329 patients and bilateral in 16 patients. Regarding multiplicity, 303 patients had a single tumor, 28 had two tumors, and 14 had more than two tumors. The parotid tumors were resected via superficial parotidectomy (*n* = 316; 91%). Total parotidectomy was performed in 31 patients with deep-lobe tumors. The mean tumor diameter was 2.5 cm. The patient demographic data are summarized in Table 1.

**Table 1:** Demographic characteristics of parotid tumor patients

Age, mean (range), y	55.7 (14–85)
Sex, No. (%)	
Male	197 (56)
Female	150 (43)
Location, No. (%)	
Superficial lobe	208 (60)
Deep lobe	27 (8)
Whole lobe	112 (32)
Laterality, No. (%)	
Unilateral	329 (95)
Bilateral	16 (5)
Multiplicity, No. (%)	
Single	303 (87)
Two	28 (8)
More than two	14 (4)
Operation, No. (%)	
Partial parotidectomy	316 (91)
Total parotidectomy	31 (9)
Mean diameter, cm	2.5

### FNA results and final diagnosis distribution

FNA revealed 318 (92%) benign and 10 (3%) malignant tumors. Nineteen specimens (5%) were unsatisfactory for evaluation because of insufficient cells or excessive artifacts. The distribution of the cytological diagnoses of parotid lesions is summarized in Table 2.

**Table 2:** Fine-needle aspiration cytology (FNAC) distribution of parotid lesions

Cytologic Diagnosis	No. (%)
**Benign**	**318 (92)**
Pleomorphic adenoma	120
Warthin tumor	77
Basal cell adenoma	2
Lymph node	1
Abscess	2
Negative for malignancy	30
Other benign	86
**Malignant**	**10 (3)**
Mucoepidermoid carcinoma	3
Adenoid cystic carcinoma	2
Other Malignant	5
**Unsatisfactory**	**19 (5)**

The final histological diagnoses included 303 (87%) benign and 44 (13%) malignant lesions. The detailed histopathological data are shown in Table 3.

**Table 3:** Histologic types distribution of parotid lesions

Histologic Diagnosis	No. (%)
**Benign**	**303 (87)**
Pleomorphic adenoma	134
Warthin tumor	122
Basal cell adenoma	9
Oncocytoma	4
Lymph node	4
Abscess	2
Other benign	28
**Malignant**	**44 (13)**
Mucoepidermoid carcinoma	15
Carcinoma ex pleomorphic adenoma	5
Acinic cell carcinoma	5
Salivary duct carcinoma	4
Adenoid cystic carcinoma	4
Epithelial myoepithelial carcinoma	3
Lymphoma	3
Basal cell adenocarcinoma	2
Other Malignant	3

### Accuracy of fine-needle aspiration cytology

When calculating sensitivity, specificity, accuracy, positive predictive value, and negative predictive value, we categorized tumor types based on benign and malignant cells rather than specific tumor types. The sensitivity, specificity, accuracy, positive predictive value (PPV), and negative predictive value (NPV) of FNA were 20%, 99%, 89%, 90% and 89%, respectively. (Table 4). When considering the specific type of tumor, preoperative FNA and final histopathological findings were concordant in 185 (53%) patients. There was concordance between the FNA results and final biopsy results in distinguishing between benign and malignant tumors in 108 (31%) patients, despite the different types of tumors. In contrast, in 34 patients, preoperative FNA examinations indicated benign findings, whereas postoperative histopathological evaluations revealed malignancy. Conversely, in 2 patients, FNA was suspicious of malignancy, but a postoperative final biopsy confirmed a benign diagnosis. Among the 337 patients with benign or unsatisfactory results on preoperative FNA related to parotid tumors, 36 were subsequently diagnosed as malignant, resulting in a false-negative rate of 10.6%.

**Table 4:** Accuracy of parotid gland fine-needle aspiration cytology

Cytologic Impression	Sensitivity	Specificity	Accuracy	PPV	NPV
Malignant or Suspicious (%)	20	99	89	90	89

Abbreviations: PPV=positive predictive value, NPV=negative predictive value

### Imaging findings

The true-negative group consisted of 301 patients who had benign or unsatisfactory results on preoperative FNA and were confirmed to be benign on the final histological examination. The false-negative group comprised 36 patients who had benign or unsatisfactory results on FNA but were diagnosed with malignancy on the final histological examination.

A logistic regression analysis was conducted to identify factors associated with malignancy in patients with benign or unsatisfactory FNA results. The univariate analysis identified several imaging parameters as significantly associated with malignancy, including irregular shape (Odds ratio [OR] = 5.04, 95% confidence interval [CI]: 2.46–10.31), ill-defined margins (OR = 3.99, 95% CI: 1.85–8.63), and invasion (OR = 12.02, 95% CI: 3.07–47.09) on T2-weighted MRI images. Factors with a P-value of < 0.05, as determined by univariate analysis, were included in the multivariate analysis. In the multivariate analysis, irregular shape (OR = 3.06, 95% CI: 1.16–8.04, *P* = 0.02) and invasion (OR = 12.73, 95% CI: 1.27–127.4, *P* = 0.03) remained significant, emerging as independent predictors of malignancy even when the FNA results did not indicate malignancy. In the T1 sequence, we analyzed the tumor whether it was inhomogeneous, high signal, or low signal in order to identify tumor’s characteristics. No significant statistical results were obtained from the T1 sequences. These findings highlight the importance of considering imaging characteristics, such as irregular shape and invasion) on T2-weighted MRI images, in the preoperative assessment of parotid gland tumors. (Table 5).

**Table 5:** Univariate and Multivariate logistic regression analysis for predicting the malignancy on biopsy in patients with benign or unsatisfactory fine-needle aspiration (FNA) results

Variable		Univariate analysis		Multivariate analysis	
		OR (95% CI)	*P*-value	OR (95% CI)	*P*-value
Age		0.99 (0.97–1.02)	0.66	1.01 (0.98–1.04)	0.55
Sex (ref = male)		0.65 (0.32–1.3)	0.22	0.7 (0.31–1.55)	0.37
DM		0.59 (0.2–1.73)	0.33	0.66 (0.19–2.29)	0.51
HTN		0.74 (0.35–1.55)	0.42	0.96 (0.38–2.43)	0.93
Diameter		0.98 (0.72–1.34)	0.9	0.96 (0.65–1.43)	0.85
Location (ref = Superficial)					
Deep		1.03 (0.29–3.7)	0.97	0.51 (0.11–2.39)	0.39
Whole		0.69 (0.31–1.53)	0.36	0.43 (0.16–1.2)	0.11
Multiplicity (ref = Single)					
Two		0.61 (0.14–2.69)	0.51	0.94 (0.2–4.48)	0.94
More than Two		1.5 (0.32–7.07)	0.61	3.66 (0.65–20.5)	0.14
Enhancement (ref = Homogeneous)					
Heterogeneous		2.22 (0.82–6.96)	0.12		
Subtle		0.54 (0.1–2.92)	0.48		
**Shape (ref = Regular)**					
**Irregular**		**5.04 (2.46–10.31)**	< 0.01*	**3.06 (1.16–8.04)**	**0.02**
**Margin (ref = Well-defined)**					
**Ill-defined**		**3.99 (1.85–8.63)**	< 0.01*	2.14 (0.79–5.85)	0.14
**Infiltrative**		**19.35 (3.05-122.59)**	< 0.01*	1.09 (0.06–18.63)	0.95
Cystic		0.72 (0.35–1.48)	0.37		
Lobulated		0.56 (0.26–1.21)	0.14		
**Invasion**		**12.02 (3.07–47.09)**	< 0.01*	**12.73 (1.27–127.4)**	**0.03**
Calcification	0.65 (0.18–2.37)		0.52		

## Discussion

**Fig. 1:**
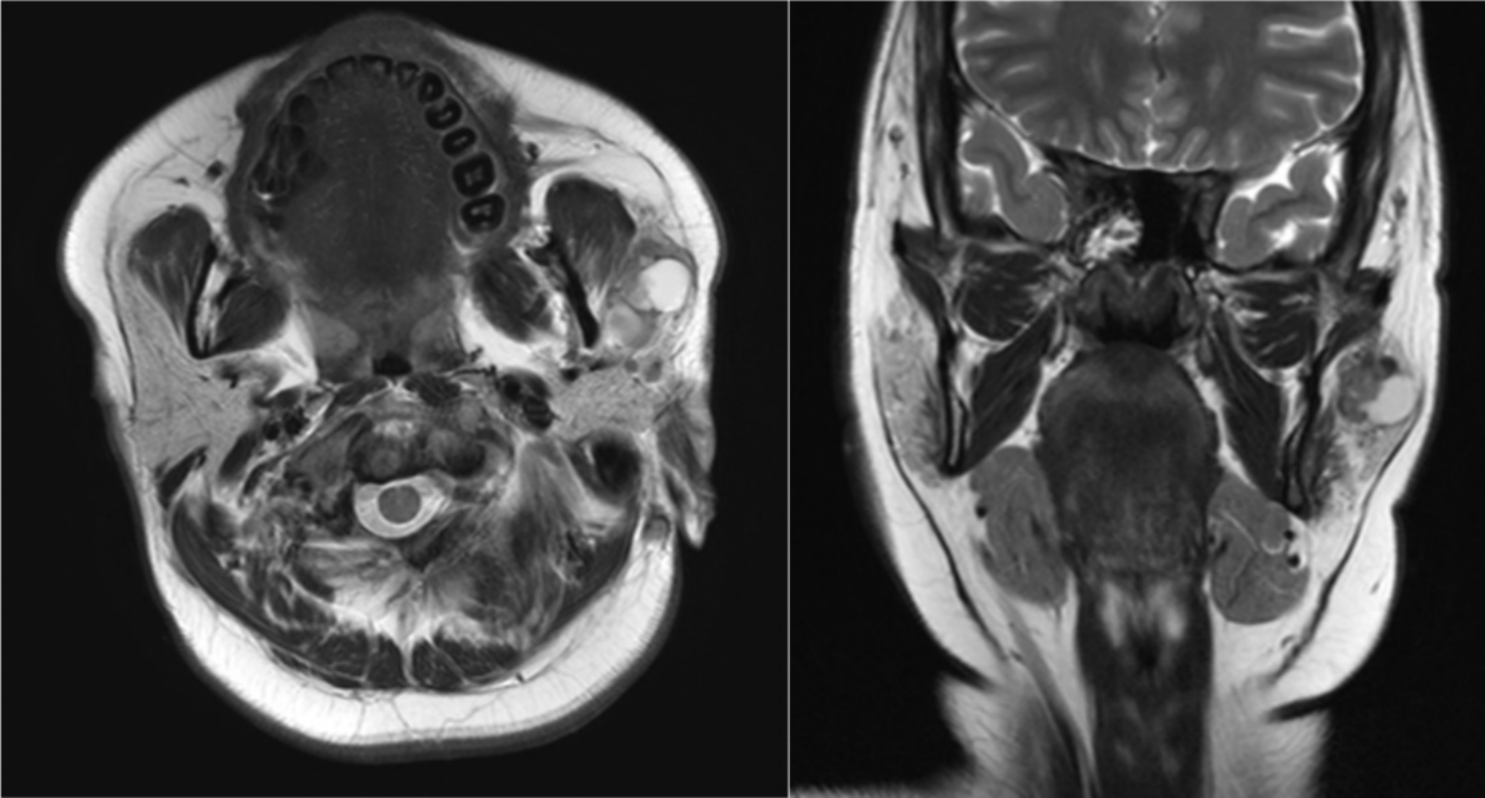
T2-weighted magnetic resonance axial and coronal imaging of a 20-year-old female. The lesion, approximately 3.0 cm in size, was a mixed solid and cystic mass located on the anterior side of the left parotid gland. It had an irregular shape with well-defined margins and no surrounding invasion. Preoperative FNA result was negative for malignancy. The patient underwent a left partial parotidectomy, and the final postoperative histopathological diagnosis was mucoepidermoid carcinoma

**Fig. 2:**
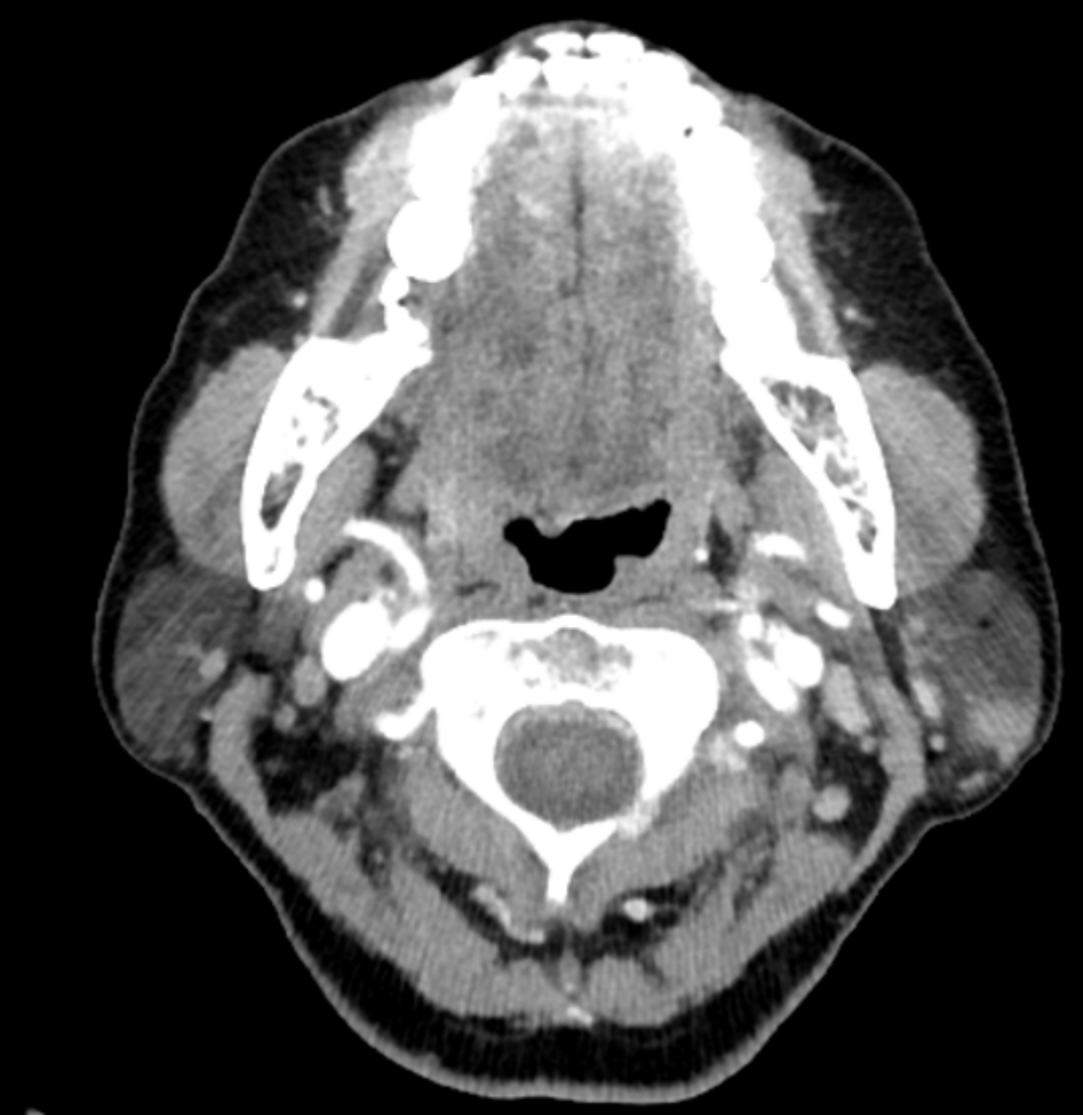
CT axial image of a 61-year-old female. An irregularly shaped, ill-defined lesion about 1.0 cm in size was identified in the superficial lobe of the left parotid gland. There was no evidence of invasion into surrounding structures. Preoperative FNA results indicated a Warthin tumor. The patient underwent a left partial parotidectomy and the final postoperative histopathological diagnosis was mucoepidermoid carcinoma

**Fig. 3:**
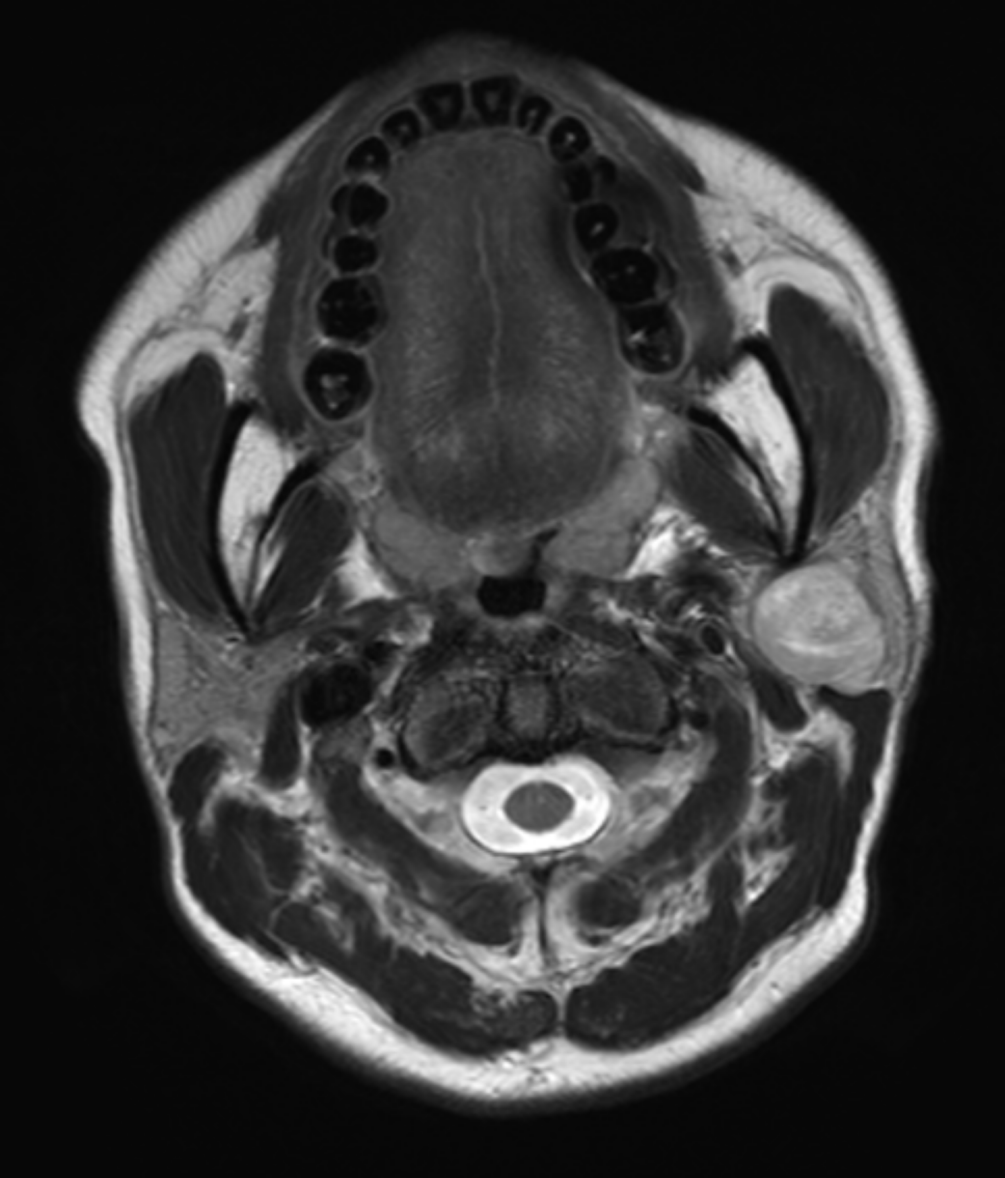
T2-weighted magnetic resonance axial imaging of a 35-year-old female. The lesion, approximately 3.6 cm cm in size, was heterogeneously enhancing mass with well-defined margin, located on the superficial and deep lobe of the left parotid gland. It had a regular shape and no surrounding invasion. Preoperative FNA result was adenoid cystic carcinoma. The patient underwent a Lt parotidectomy, and the final postoperative histopathological diagnosis was pleomorphic adenoma

In this study, we sought to evaluate typical image characteristics, specifically MRI and CT, to improve the discrimination between benign and malignant parotid tumors, particularly in cases with false-negative FNA results. Our results indicated that certain imaging characteristics, such as irregular shape and invasion were significantly associated with malignancy when the FNA results were benign or unsatisfactory. Multivariate analysis identified tumor shape and invasion as independent factors influencing diagnostic accuracy. The imaging examinations of representative patients are attached as figures. (Figure 1, 2 and 3.)

Although the literature regarding the imaging characteristics of malignant parotid tumor and their differential diagnosis from benign tumors remains limited. In our study, the shape variable yielded a significant (odds ratio, 5.04) in univariate analysis but lost its significance in multivariate logistic regression analysis. An irregular shape on MRI is often indicative of an aggressive and malignant neoplasm, whereas well-defined lesion borders are observed in low-grade malignant neoplasms and benign neoplasms [9]. From our study, ill-defined and infiltrative margins were also found to be statistically significant. Among the well-established imaging findings favoring malignancy, high-grade tumors are sometimes identified using radiological criteria, including irregular margins and infiltrative growth [9, 10]. Infiltration into deeper structures appears to be the most reliable characteristic of a malignant parotid tumor on MRI [11]. 

However, there are still undetermined characteristics of imaging features. There is ongoing controversy surrounding this enhancement in various studies. Some studies argue that tumor homogeneity is not a useful criterion for distinguishing benign from malignant disease [12, 13]. Additionally, the degree of tumor enhancement after contrast administration did not significantly help differentiate benign from malignant tumors, although there was a tendency toward strong enhancement in benign tumors [14]. Similarly, our study did not find statistical significance in the enhancement category. But some authors have reported that tumor homogeneity correlates well with histologic findings [10, 15]. In our study, the presence of cystic areas did not yield significant results. While some studies have suggested a relationship between cystic features and malignancy, mucoepidermoid carcinoma has been described as either a solid mass with cystic components (low-grade type) or an infiltrating lesion (high-grade type) [16, 17]. In contrast, other studies have argued that cystic/necrotic areas are not statistically significant for diagnosing malignant tumors because of the high prevalence of such areas in benign tumors, such as Warthin tumors or pleomorphic adenoma. In summary, definitive imaging characteristics for predicting malignant parotid tumors have not yet been established. Therefore, we conducted this study focusing on patients with false-negative results. We believe that the combined use of clinical features and preoperative imaging characteristics could effectively identify high-risk patients with malignant parotid tumor, with more aggressive management or additional evaluations, such as intraoperative frozen biopsy.

Moreover, we also obtained several insights into the diagnostic process for parotid tumors. FNA is a commonly used procedure for the preoperative assessment of these tumors and provides a safe and cost-effective means of obtaining cytological information. Fine-needle aspiration cytology (FNAC) is a cytological diagnostic method that is based on the morphology of a cell or group of cells and microparticles of a tissue that are acquired using a needle [18]. According to established guidelines, FNAC is included in the diagnostic workup for parotid gland lesions to rule out inflammation, identify systemic disease, exclude direct gland invasion or metastases in patients with an oncologic history, evaluate unresectable tumors in poor surgical candidates, and assess patients with a low risk of neoplasms [19]. However, owing to its low sensitivity, it is necessary to interpret it in the context of all other clinical information [20]. And our study confirmed that FNA results can sometimes be inadequate. We observed a false-negative rate of 10.6% in patients with benign or unsatisfactory FNA results, and subsequent histopathological evaluation revealed malignancy. Sampling errors may be a common reason for false-negative FNA findings. Malignant mixed tumors are heterogeneous lesions that frequently exhibit both benign and malignant features. Similarly, low-grade mucoepidermoid carcinomas are difficult to diagnose by cytopathological evaluation alone because of the heterogeneous cellular population and scant cellularity [21]. And classic morphologic features like necrosis, mitotic figures, and nuclear atypia, are rare or absent in acinic cell carcinomas, contributing to a relatively elevated incidence of false-negative findings. This highlights the limitations of FNA as a standalone diagnostic tool for parotid tumors and the need for complementary methods to enhance diagnostic accuracy.

This study has several limitations. First, the sample size was relatively small, especially for malignant parotid tumors. Since patients were recruited from two institutions, a larger population from multiple centers would be needed for further validation of our findings. Second, the malignant group has a variety of pathological types, which may lead to an overestimation of diagnostic accuracy. And its retrospective nature and potential selection bias, as it involved patients who underwent surgical resection, should be considered.

## Conclusion

Our study found an association with preoperative diagnosis of parotid tumors, especially in cases with false-negative FNA results. Radiological imaging, which focuses on specific characteristics, such as irregular shape and invasion, can provide valuable additional information to guide treatment planning. Furthermore, even when FNA results indicate benign lesions or are unsatisfactory, suspicion of malignancy based on imaging findings may warrant additional procedures such as frozen biopsies to ensure optimal patient care and treatment decisions.

## Data Availability

Data Availability Data available on request due to privacy/ethical restrictions. The data that support the findings of this study are available on request from the corresponding author, [Young-Gyu Eun]. The data are not publicly available due to information that could compromise the privacy of research participants.

## Appendix I. Sensitivity, specificity, accuracy, positive predictive value (PPV), and negative predictive value (NPV) of FNA

- Sensitivity = true positive / (true positive + false negative);
- Specificity = true negative / (true negative + false positive);
- Accuracy = (true positive + true negative) / total;
- PPV = true positive / (true positive + false positive);
- NPV = true negative / (true negative + false positive).
